# *N*,*N*-Bis(diphenyl­phosphanyl)cyclo­propyl­amine

**DOI:** 10.1107/S1600536810041711

**Published:** 2010-10-23

**Authors:** Ilana Engelbrecht, Hendrik G. Visser, Andreas Roodt

**Affiliations:** a Department of Chemistry, University of the Free State, PO Box 339, Bloemfontein, 9300, South Africa

## Abstract

In the title compound, C_27_H_25_NP_2_, the diphenyl­phosphino groups are staggered relative to the PNP backbone. The dihedral angles between the phenyl rings bonded to each P atom are 51.74 (5) and 68.23 (4)°. The coordination around the N atom deviates from trigonal-pyrimidal geometry towards an almost planar arrangement between the N atom and the adjacent P and C atoms; the distance between the N atom and the plane formed by the adjacent C/P/P atoms is 0.098 (2) Å.

## Related literature

For similar non-coordinated diphosphineamine ligands with distorted trigonal-pyramidal geometries see: Fei *et al.* (2003 ▶); Keat *et al.* (1981 ▶); Cotton *et al.* (1996 ▶); Cloete *et al.* (2008 ▶, 2009 ▶, 2010 ▶).
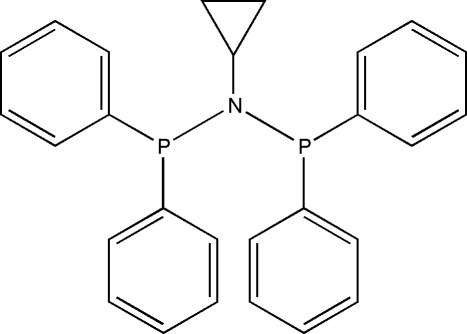


## Experimental

### 

#### Crystal data

C_27_H_25_NP_2_
*M* *_r_* = 425.42
Monoclinic, 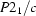

*a* = 14.460 (4) Å
*b* = 10.486 (5) Å
*c* = 17.053 (5) Å
β = 119.358 (5)°
*V* = 2253.6 (15) Å^3^
*Z* = 4
Mo *K*α radiation
μ = 0.21 mm^−1^
*T* = 100 K
0.33 × 0.18 × 0.16 mm

#### Data collection

Bruker X8 APEXII 4K Kappa CCD diffractometer
Absorption correction: multi-scan (*SADABS*; Bruker, 2004 ▶) *T*_min_ = 0.935, *T*_max_ = 0.968
33310 measured reflections
5581 independent reflections
4477 reflections with *I* > 2.0σ(*I*)
*R*_int_ = 0.041

#### Refinement

*R*[*F* ^2^ > 2σ(*F* ^2^)] = 0.039
*wR*(*F* ^2^) = 0.105
*S* = 1.06
5581 reflections
271 parameters
H-atom parameters constrained
Δρ_max_ = 0.33 e Å^−3^
Δρ_min_ = −0.36 e Å^−3^

### 

Data collection: *APEX2* (Bruker, 2010 ▶); cell refinement: *SAINT-Plus* (Bruker, 2004 ▶); data reduction: *SAINT-Plus*; program(s) used to solve structure: *SHELXS97* (Sheldrick, 2008 ▶); program(s) used to refine structure: *SHELXL97* (Sheldrick, 2008 ▶); molecular graphics: *DIAMOND* (Brandenburg & Putz, 2005 ▶); software used to prepare material for publication: *WinGX* (Farrugia, 1999 ▶).

## Supplementary Material

Crystal structure: contains datablocks global, I. DOI: 10.1107/S1600536810041711/pv2338sup1.cif

Structure factors: contains datablocks I. DOI: 10.1107/S1600536810041711/pv2338Isup2.hkl

Additional supplementary materials:  crystallographic information; 3D view; checkCIF report

## Figures and Tables

**Fig. 1.**
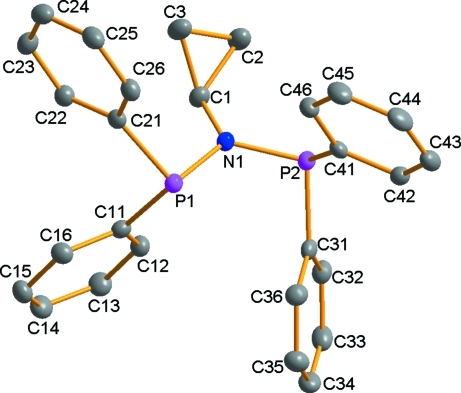
Molecular structure of the title compound. Displacement ellipsoids are drawn at the 50% probability level. Hydrogen atoms have been omitted for clarity.

## supplementary crystallographic information

### Comment

This structure forms part of ongoing research on ethylene tetramerization. In the title compound, all bond distances and angles are normal and fall within the range for similar complexes (Keat *et al.*, 1981; Cotton *et al.*, 1996; Cloete *et al.*, 2008; 2009; 2010). The distance of N1 from the P1—P2—C1 plane is 0.098 (2) Å showing that the N atom adapts a planar geometry with these atoms in order to accomodate the steric bulk of the phenyl groups. Dihedral angles were calculated based on planes defined by each phenyl ring and the neighboring P atom. The dihedral angles between the various phenyl rings within the molecule are: C1/C2 = 51.74 (5) ° and C3/C4 = 68.23 (4) °. TThe distorted tetrahedral angles of the P atoms range between 100.43 (7) and 105.26 (7) ° which is in good comparison with those in literature. There are no classical intermolecular hydrogen interactions. The title compound is a C_s_ conformer in the solid state (Keat *et al.*, 1981) in which the phosphorous lone pairs are *trans* with respect to the N–C bond.

### Experimental

Cyclopropylamine (0.010 mol, 693 µl) was dissolved in dichloromethane (30 ml) after which the solution was placed on an ice bath. Triethylamine (0.030 mol, 4.21 ml) was added to the solution while stirring. Chlorodiphenylphosphine (0.020 mol, 3.70 ml) was slowly added to the reaction mixture. The ice bath was removed after 1 h and the reaction mixture was allowed to stir at room temperature for a further 13 h. The dichloromethane was removed under reduced pressure. A mixture of hexane (20 ml) and toluene (2 ml) was added to the remaining white powder and was passed through a column containing neutral activated alumina (35 g). The solvent of the eluent was removed under reduced pressure and the white precipitate was collected. Single colourless crystals suitable for X-ray crystallography were obtained from recrystallization from methanol. (yield: 1.807 g, 43%)

### Refinement

The methine, methylene and aromatic H atoms were placed in geometrically idealized positions at C—H = 1.00, 0.99 and 0.95 Å, respectively and constrained to ride on their parent atoms, with *U*_iso_(H) = 1.2*U*_eq_(C). The highest peak is located 0.71 Å from C43 and the deepest hole is situated 0.53 Å from P1.

### Crystal data

**Table:**

C_27_H_25_NP_2_	*F*(000) = 896
*M**_r_* = 425.42	*D*_x_ = 1.254 Mg m^−^^3^
Monoclinic, *P*2_1_/*c*	Mo *K*α radiation, λ = 0.71069 Å
Hall symbol: -P 2ybc	Cell parameters from 8432 reflections
*a* = 14.460 (4) Å	θ = 2.5–27.5°
*b* = 10.486 (5) Å	µ = 0.21 mm^−^^1^
*c* = 17.053 (5) Å	*T* = 100 K
β = 119.358 (5)°	Cuboid, colourless
*V* = 2253.6 (15) Å^3^	0.33 × 0.18 × 0.16 mm
*Z* = 4	

### Data collection

**Table:**

Bruker X8 APEXII 4K Kappa CCD diffractometer	5581 independent reflections
Radiation source: fine-focus sealed tube	4477 reflections with *I* > 2.0σ(*I*)
graphite	*R*_int_ = 0.041
ω and φ scans	θ_max_ = 28.4°, θ_min_ = 3.2°
Absorption correction: multi-scan (*SADABS*; Bruker, 2004)	*h* = −18→19
*T*_min_ = 0.935, *T*_max_ = 0.968	*k* = −10→13
33310 measured reflections	*l* = −22→22

### Refinement

**Table:**

Refinement on *F*^2^	Primary atom site location: structure-invariant direct methods
Least-squares matrix: full	Secondary atom site location: difference Fourier map
*R*[*F*^2^ > 2σ(*F*^2^)] = 0.039	Hydrogen site location: inferred from neighbouring sites
*wR*(*F*^2^) = 0.105	H-atom parameters constrained
*S* = 1.06	*w* = 1/[σ^2^(*F*_o_^2^) + (0.0466*P*)^2^ + 1.0215*P*] where *P* = (*F*_o_^2^ + 2*F*_c_^2^)/3
5581 reflections	(Δ/σ)_max_ < 0.001
271 parameters	Δρ_max_ = 0.33 e Å^−^^3^
0 restraints	Δρ_min_ = −0.36 e Å^−^^3^

### Special details

**Table:**

Experimental. The intensity data were collected on a Bruker X8 ApexII 4 K Kappa CCD diffractometer using an exposure time of 10 s/frame. A total of 1407 frames were collected with a frame width of 0.5° covering up to θ = 28.36° with 99.3% completeness accomplished. Spectroscopy data: ^1^H NMR (300 MHz, CD_2_Cl_2_): δ = 0.3 to 0.7 (m, 4H, 2 *x* CH_2_), 2.6 (m, 1H, CH), 7.3 to 7.5 (m, 20H, Ar); ^31^P NMR (121 MHz, CD_2_Cl_2_): δ = 63.4 (*s*).
Geometry. All s.u.'s (except the s.u. in the dihedral angle between two l.s. planes) are estimated using the full covariance matrix. The cell s.u.'s are taken into account individually in the estimation of s.u.'s in distances, angles and torsion angles; correlations between s.u.'s in cell parameters are only used when they are defined by crystal symmetry. An approximate (isotropic) treatment of cell s.u.'s is used for estimating s.u.'s involving l.s. planes.
Refinement. Refinement of *F*^2^ against ALL reflections. The weighted *R*-factor *wR* and goodness of fit *S* are based on *F*^2^, conventional *R*-factors *R* are based on *F*, with *F* set to zero for negative *F*^2^. The threshold expression of *F*^2^ > 2σ(*F*^2^) is used only for calculating *R*-factors(gt) *etc*. and is not relevant to the choice of reflections for refinement. *R*-factors based on *F*^2^ are statistically about twice as large as those based on *F*, and *R*- factors based on ALL data will be even larger.

### Fractional atomic coordinates and isotropic or equivalent isotropic displacement parameters (Å^2^)

**Table:**

	*x*	*y*	*z*	*U*_iso_*/*U*_eq_	
C1	0.59558 (12)	0.25225 (15)	0.55966 (10)	0.0209 (3)	
H1	0.5473	0.1995	0.5729	0.025*	
C2	0.54277 (14)	0.32353 (16)	0.47190 (11)	0.0267 (4)	
H2A	0.4654	0.3121	0.4327	0.032*	
H2B	0.5833	0.3354	0.4396	0.032*	
C3	0.58012 (14)	0.39375 (16)	0.55920 (11)	0.0274 (4)	
H3A	0.6436	0.4488	0.5804	0.033*	
H3B	0.5256	0.4255	0.5735	0.033*	
C11	0.71357 (12)	0.07960 (14)	0.74371 (10)	0.0184 (3)	
C12	0.63529 (12)	−0.00544 (14)	0.68578 (11)	0.0211 (3)	
H12	0.6151	−0.0062	0.6237	0.025*	
C13	0.58661 (13)	−0.08882 (16)	0.71768 (11)	0.0248 (3)	
H13	0.5327	−0.145	0.6773	0.03*	
C14	0.61675 (14)	−0.08999 (16)	0.80836 (12)	0.0258 (4)	
H14	0.5834	−0.1467	0.8303	0.031*	
C15	0.69601 (13)	−0.00792 (15)	0.86719 (11)	0.0245 (3)	
H15	0.7173	−0.0093	0.9295	0.029*	
C16	0.74413 (13)	0.07601 (15)	0.83527 (11)	0.0213 (3)	
H16	0.7984	0.1316	0.876	0.026*	
C21	0.77084 (12)	0.33887 (14)	0.75153 (10)	0.0180 (3)	
C22	0.68675 (13)	0.37085 (15)	0.76614 (10)	0.0205 (3)	
H22	0.635	0.3084	0.7576	0.025*	
C23	0.67822 (14)	0.49346 (15)	0.79308 (11)	0.0251 (3)	
H23	0.6203	0.5145	0.8022	0.03*	
C24	0.75390 (14)	0.58542 (16)	0.80678 (11)	0.0258 (4)	
H24	0.7471	0.6694	0.8242	0.031*	
C25	0.83918 (14)	0.55410 (15)	0.79498 (11)	0.0241 (3)	
H25	0.8921	0.616	0.8057	0.029*	
C26	0.84747 (13)	0.43169 (15)	0.76732 (10)	0.0207 (3)	
H26	0.9061	0.4109	0.7591	0.025*	
C31	0.76517 (12)	−0.03248 (14)	0.55113 (10)	0.0182 (3)	
C32	0.68867 (13)	−0.12527 (15)	0.50250 (11)	0.0222 (3)	
H32	0.6234	−0.1006	0.4519	0.027*	
C33	0.70678 (14)	−0.25267 (15)	0.52714 (11)	0.0262 (4)	
H33	0.654	−0.3147	0.4938	0.031*	
C34	0.80162 (14)	−0.28923 (15)	0.60026 (12)	0.0272 (4)	
H34	0.8142	−0.3766	0.6168	0.033*	
C35	0.87866 (14)	−0.19906 (15)	0.64963 (12)	0.0269 (4)	
H35	0.9436	−0.2245	0.7002	0.032*	
C36	0.86059 (13)	−0.07105 (15)	0.62481 (11)	0.0223 (3)	
H36	0.9137	−0.0095	0.6583	0.027*	
C41	0.85308 (12)	0.20398 (14)	0.53650 (10)	0.0173 (3)	
C42	0.91036 (12)	0.14796 (15)	0.49881 (10)	0.0201 (3)	
H42	0.8889	0.0675	0.4699	0.024*	
C43	0.99796 (13)	0.20875 (16)	0.50336 (11)	0.0239 (3)	
H43	1.0358	0.1699	0.4772	0.029*	
C44	1.03073 (13)	0.32609 (16)	0.54587 (12)	0.0267 (4)	
H44	1.0911	0.3672	0.5492	0.032*	
C45	0.97516 (14)	0.38290 (15)	0.58346 (12)	0.0266 (4)	
H45	0.9978	0.4628	0.6131	0.032*	
C46	0.88620 (13)	0.32314 (14)	0.57785 (11)	0.0222 (3)	
H46	0.8474	0.3638	0.6024	0.027*	
N1	0.70166 (10)	0.20116 (11)	0.59277 (8)	0.0171 (3)	
P1	0.78650 (3)	0.18492 (4)	0.70717 (3)	0.01690 (10)	
P2	0.72688 (3)	0.13260 (4)	0.51349 (3)	0.01718 (10)	

### Atomic displacement parameters (Å^2^)

**Table:**

	*U*^11^	*U*^22^	*U*^33^	*U*^12^	*U*^13^	*U*^23^
C1	0.0195 (7)	0.0222 (8)	0.0204 (8)	0.0035 (6)	0.0094 (6)	−0.0003 (6)
C2	0.0286 (9)	0.0274 (8)	0.0215 (8)	0.0078 (7)	0.0101 (7)	0.0011 (7)
C3	0.0304 (9)	0.0247 (8)	0.0227 (8)	0.0083 (7)	0.0097 (7)	0.0006 (7)
C11	0.0196 (7)	0.0158 (7)	0.0213 (8)	0.0023 (6)	0.0112 (6)	0.0005 (6)
C12	0.0254 (8)	0.0202 (7)	0.0193 (8)	−0.0002 (6)	0.0124 (7)	−0.0017 (6)
C13	0.0272 (9)	0.0216 (8)	0.0261 (9)	−0.0058 (6)	0.0135 (7)	−0.0045 (7)
C14	0.0304 (9)	0.0222 (8)	0.0306 (9)	−0.0029 (7)	0.0194 (8)	0.0013 (7)
C15	0.0301 (9)	0.0257 (8)	0.0203 (8)	0.0007 (7)	0.0142 (7)	0.0019 (6)
C16	0.0227 (8)	0.0202 (7)	0.0196 (8)	−0.0014 (6)	0.0092 (6)	−0.0003 (6)
C21	0.0208 (8)	0.0186 (7)	0.0134 (7)	0.0010 (6)	0.0073 (6)	0.0002 (6)
C22	0.0220 (8)	0.0209 (7)	0.0187 (8)	−0.0005 (6)	0.0102 (6)	−0.0007 (6)
C23	0.0283 (9)	0.0251 (8)	0.0244 (8)	0.0051 (7)	0.0148 (7)	−0.0007 (7)
C24	0.0362 (9)	0.0186 (7)	0.0209 (8)	0.0025 (7)	0.0127 (7)	−0.0009 (6)
C25	0.0315 (9)	0.0206 (8)	0.0186 (8)	−0.0048 (7)	0.0112 (7)	−0.0011 (6)
C26	0.0227 (8)	0.0229 (8)	0.0165 (7)	−0.0022 (6)	0.0097 (6)	−0.0011 (6)
C31	0.0225 (8)	0.0173 (7)	0.0216 (8)	−0.0009 (6)	0.0161 (7)	−0.0015 (6)
C32	0.0253 (8)	0.0222 (8)	0.0232 (8)	−0.0029 (6)	0.0150 (7)	−0.0034 (6)
C33	0.0365 (10)	0.0211 (8)	0.0279 (9)	−0.0086 (7)	0.0210 (8)	−0.0066 (7)
C34	0.0413 (10)	0.0162 (7)	0.0311 (9)	−0.0017 (7)	0.0233 (8)	0.0003 (7)
C35	0.0289 (9)	0.0238 (8)	0.0286 (9)	0.0024 (7)	0.0146 (7)	0.0052 (7)
C36	0.0226 (8)	0.0192 (7)	0.0270 (8)	−0.0018 (6)	0.0136 (7)	−0.0008 (6)
C41	0.0200 (7)	0.0178 (7)	0.0147 (7)	0.0011 (6)	0.0088 (6)	0.0038 (6)
C42	0.0241 (8)	0.0178 (7)	0.0198 (8)	0.0027 (6)	0.0118 (7)	0.0018 (6)
C43	0.0246 (8)	0.0263 (8)	0.0264 (8)	0.0054 (6)	0.0169 (7)	0.0078 (7)
C44	0.0248 (8)	0.0280 (8)	0.0291 (9)	−0.0039 (7)	0.0147 (7)	0.0077 (7)
C45	0.0343 (9)	0.0196 (8)	0.0255 (9)	−0.0068 (7)	0.0143 (8)	0.0001 (6)
C46	0.0308 (9)	0.0178 (7)	0.0224 (8)	−0.0005 (6)	0.0164 (7)	0.0010 (6)
N1	0.0179 (6)	0.0186 (6)	0.0155 (6)	0.0020 (5)	0.0088 (5)	−0.0004 (5)
P1	0.0173 (2)	0.01718 (19)	0.0168 (2)	0.00046 (14)	0.00886 (16)	−0.00058 (14)
P2	0.0189 (2)	0.01685 (19)	0.0170 (2)	0.00047 (14)	0.00976 (16)	−0.00097 (14)

### Geometric parameters (Å, °)

**Table:**

C1—N1	1.452 (2)	C25—C26	1.393 (2)
C1—C3	1.500 (2)	C25—H25	0.95
C1—C2	1.504 (2)	C26—H26	0.95
C1—H1	1	C31—C36	1.395 (2)
C2—C3	1.504 (2)	C31—C32	1.399 (2)
C2—H2A	0.99	C31—P2	1.8349 (17)
C2—H2B	0.99	C32—C33	1.386 (2)
C3—H3A	0.99	C32—H32	0.95
C3—H3B	0.99	C33—C34	1.380 (3)
C11—C12	1.399 (2)	C33—H33	0.95
C11—C16	1.399 (2)	C34—C35	1.387 (2)
C11—P1	1.8342 (16)	C34—H34	0.95
C12—C13	1.390 (2)	C35—C36	1.393 (2)
C12—H12	0.95	C35—H35	0.95
C13—C14	1.386 (2)	C36—H36	0.95
C13—H13	0.95	C41—C46	1.399 (2)
C14—C15	1.390 (2)	C41—C42	1.402 (2)
C14—H14	0.95	C41—P2	1.8282 (16)
C15—C16	1.388 (2)	C42—C43	1.386 (2)
C15—H15	0.95	C42—H42	0.95
C16—H16	0.95	C43—C44	1.388 (2)
C21—C22	1.396 (2)	C43—H43	0.95
C21—C26	1.398 (2)	C44—C45	1.384 (2)
C21—P1	1.8433 (17)	C44—H44	0.95
C22—C23	1.391 (2)	C45—C46	1.391 (2)
C22—H22	0.95	C45—H45	0.95
C23—C24	1.390 (2)	C46—H46	0.95
C23—H23	0.95	N1—P2	1.7208 (13)
C24—C25	1.383 (2)	N1—P1	1.7301 (14)
C24—H24	0.95		
			
N1—C1—C3	119.55 (14)	C26—C25—H25	120
N1—C1—C2	119.64 (14)	C25—C26—C21	121.00 (15)
C3—C1—C2	60.09 (11)	C25—C26—H26	119.5
N1—C1—H1	115.5	C21—C26—H26	119.5
C3—C1—H1	115.5	C36—C31—C32	118.53 (14)
C2—C1—H1	115.5	C36—C31—P2	125.74 (12)
C1—C2—C3	59.83 (11)	C32—C31—P2	115.68 (12)
C1—C2—H2A	117.8	C33—C32—C31	120.89 (16)
C3—C2—H2A	117.8	C33—C32—H32	119.6
C1—C2—H2B	117.8	C31—C32—H32	119.6
C3—C2—H2B	117.8	C34—C33—C32	119.85 (15)
H2A—C2—H2B	114.9	C34—C33—H33	120.1
C1—C3—C2	60.07 (11)	C32—C33—H33	120.1
C1—C3—H3A	117.8	C33—C34—C35	120.37 (15)
C2—C3—H3A	117.8	C33—C34—H34	119.8
C1—C3—H3B	117.8	C35—C34—H34	119.8
C2—C3—H3B	117.8	C34—C35—C36	119.81 (16)
H3A—C3—H3B	114.9	C34—C35—H35	120.1
C12—C11—C16	118.33 (14)	C36—C35—H35	120.1
C12—C11—P1	123.02 (12)	C35—C36—C31	120.55 (15)
C16—C11—P1	118.40 (12)	C35—C36—H36	119.7
C13—C12—C11	120.96 (15)	C31—C36—H36	119.7
C13—C12—H12	119.5	C46—C41—C42	118.17 (14)
C11—C12—H12	119.5	C46—C41—P2	122.20 (12)
C14—C13—C12	119.98 (15)	C42—C41—P2	118.81 (12)
C14—C13—H13	120	C43—C42—C41	120.72 (15)
C12—C13—H13	120	C43—C42—H42	119.6
C13—C14—C15	119.78 (15)	C41—C42—H42	119.6
C13—C14—H14	120.1	C42—C43—C44	120.36 (15)
C15—C14—H14	120.1	C42—C43—H43	119.8
C16—C15—C14	120.26 (15)	C44—C43—H43	119.8
C16—C15—H15	119.9	C45—C44—C43	119.75 (15)
C14—C15—H15	119.9	C45—C44—H44	120.1
C15—C16—C11	120.65 (15)	C43—C44—H44	120.1
C15—C16—H16	119.7	C44—C45—C46	120.09 (15)
C11—C16—H16	119.7	C44—C45—H45	120
C22—C21—C26	118.40 (14)	C46—C45—H45	120
C22—C21—P1	125.20 (12)	C45—C46—C41	120.89 (15)
C26—C21—P1	116.33 (12)	C45—C46—H46	119.6
C23—C22—C21	120.45 (15)	C41—C46—H46	119.6
C23—C22—H22	119.8	C1—N1—P2	116.03 (10)
C21—C22—H22	119.8	C1—N1—P1	120.30 (10)
C24—C23—C22	120.45 (16)	P2—N1—P1	122.58 (8)
C24—C23—H23	119.8	N1—P1—C11	103.56 (7)
C22—C23—H23	119.8	N1—P1—C21	102.40 (7)
C25—C24—C23	119.71 (15)	C11—P1—C21	100.43 (7)
C25—C24—H24	120.1	N1—P2—C41	103.54 (7)
C23—C24—H24	120.1	N1—P2—C31	105.26 (7)
C24—C25—C26	119.94 (15)	C41—P2—C31	102.29 (7)
C24—C25—H25	120		
			
N1—C1—C2—C3	−109.10 (16)	C44—C45—C46—C41	1.6 (2)
N1—C1—C3—C2	109.25 (16)	C42—C41—C46—C45	−1.6 (2)
C16—C11—C12—C13	2.0 (2)	P2—C41—C46—C45	−171.14 (12)
P1—C11—C12—C13	176.10 (12)	C3—C1—N1—P2	−111.08 (14)
C11—C12—C13—C14	−1.1 (2)	C2—C1—N1—P2	−40.77 (18)
C12—C13—C14—C15	−0.3 (3)	C3—C1—N1—P1	80.49 (16)
C13—C14—C15—C16	0.7 (3)	C2—C1—N1—P1	150.80 (12)
C14—C15—C16—C11	0.2 (2)	C1—N1—P1—C11	58.57 (13)
C12—C11—C16—C15	−1.5 (2)	P2—N1—P1—C11	−109.08 (9)
P1—C11—C16—C15	−175.91 (12)	C1—N1—P1—C21	−45.54 (13)
C26—C21—C22—C23	1.9 (2)	P2—N1—P1—C21	146.81 (9)
P1—C21—C22—C23	−174.81 (12)	C12—C11—P1—N1	24.39 (14)
C21—C22—C23—C24	−0.7 (2)	C16—C11—P1—N1	−161.48 (12)
C22—C23—C24—C25	−1.1 (2)	C12—C11—P1—C21	129.99 (13)
C23—C24—C25—C26	1.6 (2)	C16—C11—P1—C21	−55.87 (13)
C24—C25—C26—C21	−0.3 (2)	C22—C21—P1—N1	79.58 (14)
C22—C21—C26—C25	−1.4 (2)	C26—C21—P1—N1	−97.23 (12)
P1—C21—C26—C25	175.60 (12)	C22—C21—P1—C11	−26.95 (15)
C36—C31—C32—C33	0.4 (2)	C26—C21—P1—C11	156.23 (12)
P2—C31—C32—C33	−177.16 (12)	C1—N1—P2—C41	131.82 (11)
C31—C32—C33—C34	−0.4 (2)	P1—N1—P2—C41	−60.04 (10)
C32—C33—C34—C35	0.5 (3)	C1—N1—P2—C31	−121.16 (11)
C33—C34—C35—C36	−0.6 (3)	P1—N1—P2—C31	46.98 (10)
C34—C35—C36—C31	0.6 (2)	C46—C41—P2—N1	−25.59 (14)
C32—C31—C36—C35	−0.5 (2)	C42—C41—P2—N1	164.95 (12)
P2—C31—C36—C35	176.77 (12)	C46—C41—P2—C31	−134.83 (13)
C46—C41—C42—C43	0.6 (2)	C42—C41—P2—C31	55.71 (13)
P2—C41—C42—C43	170.51 (12)	C36—C31—P2—N1	−71.90 (15)
C41—C42—C43—C44	0.4 (2)	C32—C31—P2—N1	105.44 (12)
C42—C43—C44—C45	−0.4 (2)	C36—C31—P2—C41	36.03 (15)
C43—C44—C45—C46	−0.6 (3)	C32—C31—P2—C41	−146.64 (12)
